# Therapeutic Strategies for IVD Regeneration through Hyaluronan/SDF-1-Based Hydrogel and Intravenous Administration of MSCs

**DOI:** 10.3390/ijms22179609

**Published:** 2021-09-04

**Authors:** Carla Cunha, Catarina Leite Pereira, Joana R. Ferreira, Cláudia Ribeiro-Machado, Sibylle Grad, Susana G. Santos, Raquel M. Gonçalves

**Affiliations:** 1 i3S—Instituto de Investigação e Inovação em Saúde, Universidade do Porto, 4200-135 Porto, Portugal; catarina.pereira@ineb.up.pt (C.L.P.); joana.ferreira@i3s.up.pt (J.R.F.); claudia.machado@i3s.up.pt (C.R.-M.); susana.santos@ineb.up.pt (S.G.S.); raquelg@ineb.up.pt (R.M.G.); 2 INEB—Instituto de Engenharia Biomédica, Universidade do Porto, 4200-135 Porto, Portugal; 3 ICBAS—Instituto de Ciências Biomédicas Abel Salazar, Universidade do Porto, 4050-313 Porto, Portugal; 4 AO Research Institute Davos, 7270 Davos, Switzerland; sibylle.grad@aofoundation.org

**Keywords:** intervertebral disc, MSCs, recruitment, biomaterials, inflammation

## Abstract

Intervertebral disc (IVD) degeneration involves a complex cascade of events, including degradation of the native extracellular matrix, loss of water content, and decreased cell numbers. Cell recruitment strategies for the IVD have been increasingly explored, aiming to recruit either endogenous or transplanted cells. This study evaluates the IVD therapeutic potential of a chemoattractant delivery system (HAPSDF5) that combines a hyaluronan-based thermoreversible hydrogel (HAP) and the chemokine stromal cell derived factor-1 (SDF-1). HAPSDF5 was injected into the IVD and was combined with an intravenous injection of mesenchymal stem/stromal cells (MSCs) in a pre-clinical in vivo IVD lesion model. The local and systemic effects were evaluated two weeks after treatment. The hydrogel by itself (HAP) did not elicit any adverse effect, showing potential to be administrated by intradiscal injection. HAPSDF5 induced higher cell numbers, but no evidence of IVD regeneration was observed. MSCs systemic injection seemed to exert a role in IVD regeneration to some extent through a paracrine effect, but no synergies were observed when HAPSDF5 was combined with MSCs. Overall, this study shows that although the injection of chemoattractant hydrogels and MSC recruitment are feasible approaches for IVD, IVD regeneration using this strategy needs to be further explored before successful clinical translation.

## 1. Introduction

A critical hallmark of the intervertebral disc (IVD) degenerative process is the decrease in cell viability and function, with a substantial proportion of cells existing in a senescent state [1,2]. This leads to an unbalanced matrix synthesis, the accumulation of waste products, and ultimately, to the failure of the biological and biomechanical function of the IVD [3].

In recent years, promoting cell recruitment of endogenous cell populations for the IVD has been explored as a potential therapeutic strategy [4,5]. Rather than cell transplantation, this strategy proposes the use of chemokines to enhance cell recruitment, avoiding in vitro manipulation. This may be done in combination with biomaterials, or not, and it has been revealed to be a promising approach for clinical translation compared to cell therapy [6]. In IVD, this concept has been poorly explored. Henriksson et al. demonstrated the presence of stem cell niches in rabbit IVD surroundings and migration routes from these niches towards the annulus fibrosus (AF) and the inner parts of the IVD, suggesting endogenous repair mechanisms through cell recruitment [7]. Ex vivo, Illien-Junger et al. were the first to report that mesenchymal stem/stromal cell (MSCs) migration was triggered upon IVD induced degeneration ex vivo, indicating that during the degenerative process, this tissue releases chemotactic factors that induce cell migration [8].

MSCs have a unique potential in cell-based therapies and are known by their pivotal role in the repair of several degenerative tissues, holding great potential in IVD treatment [9]. MSCs have been shown to differentiate into disc-like cells, to indirectly impact tissue regeneration through the secretion of growth factors, to act as immunomodulatory agents in the control of the IVD pro-inflammatory microenvironment, and have already been used in clinical trials, leading to an improvement in pain and disability scores [10]. MSCs are also known by their ability to migrate to sites of injury/inflammation [11], acting through differentiation into resident cells or through the secretion of paracrine factors for the reactivation of resident cells [12]. Reports on the MSCs “secretome” have contributed further to the theory that MSCs exert their function through the release of immunosuppressive factors, cytokines, growth, and differentiation factors [13,14,15]. Evidence of MSC recruitment in vivo towards a degenerative IVD has been provided by Sakai et al. using a mouse model, although a limited number of cells were found locally [16]. Finally, our own group has demonstrated that MSC recruitment towards IVD ex vivo can be enhanced using a hyaluronan-based delivery system (HAPSDF5) containing the potent chemoattractant, stromal cell derived factor-1 (SDF-1) [17]. HAPSDF5 was able to significantly increase MSC migration in IVD ex vivo and accelerate the regenerative effects of MSCs, which were previously described in the same model [18], namely by promoting faster and more pronounced collagen type II expression [19]. In addition, we have also shown that the systemic injection of MSCs in vivo in a rat IVD lesion model reduced IVD herniation. Moreover, MSC transplantation resulted in local downregulation of the hypoxia responsive GLUT-1 and in a systemic immunoregulatory effect given by the increase in MHC class II+ and CD4+ cells, the upregulation of the cytokines interleukin (IL)-2 (IL-2), IL-4, IL-6, and IL-10, and the downregulation of the cytokines IL-13 and tumor necrosis factor-α (TNF-α) [20].

Cell-based therapies have largely been explored for IVD degeneration treatment as a way to repopulate this tissue with viable cells that are capable of de novo extracellular matrix (ECM) synthesis and the activation of the endogenous cells through the release of paracrine factors. Chemoattractors, such as SDF-1, can orchestrate the directional migration of a wide spectrum of cells, including MSCs, for selected tissues [21,22]. SDF-1 specifically binds to the C-X-C chemokine receptor type 4 (CXCR4 )receptor to constitute the SDF-1/CXCR4 axis, mostly known for its role on haematopoiesis, but it has also been described as playing a key role in the repair mechanisms of different tissues [23,24]. SDF-1 and CXCR4 expression have been reported to be upregulated in degenerated human discs, particularly in the cartilage endplate (CEP) and in the nucleus pulposus (NP) tissue [25]. Moreover, the overexpression of CXCR4 in MSCs promoted the retention of these cells in IVD tissue upon transplantation [26]. The SDF-1/CXCR4 axis was implicated in the proliferation of NP cells stimulated by CEP stem cells in a paracrine way by the activation of the ERK1/2 signalling transduction pathway [27]. Although some research has been performed in cell recruitment to the IVD using in vitro cell culture and organotypic models, proof-of-concept studies in vivo are lacking. In vivo, Zhang et al. demonstrated that SDF-1 injection into rat NP resulted in dense areas of proteoglycan matrix and enhanced NP regeneration [28]; they also used albumin/heparin nanoparticles as injectable carriers of SDF-1, showing increased expression of SOX9, aggrecan, and collagen type II both at the mRNA and protein levels in a rat model [29].

Hydrogels for IVD regeneration have been extensively described. These biomaterials can mimic the hydrogel-like native NP either by mimicking NP mechanical properties, which will allow them to withstand the compressive load required for NP, or by supporting cell survival and matrix production [6]. HAP (hyaluronan and poly(*N*-iso-propylacrylamide) is a thermoreversible hydrogel composed of hyaluronan (HA), a key ECM component, and poly(*N*-isopropylacrylamide), which results in an injectable co-polymer that rapidly undergoes gelification in situ (>30 °C) [30]. HAP was previously demonstrated to support human MSC differentiation towards the NP cell phenotype (higher GAG, Col2, Sox9, KRT19, CD24, and FOXF1) [31] and has also been shown to support NP cell delivery into an ex vivo organ culture model, increasing proteoglycan content [32]. Moreover, the potential of HAP in vivo has been addressed in an osteochondral defect in the rabbit diarthrodial joint [33].

Given the promising results of the intravenous injection of MSCs in IVD herniation [20] and the potential of HAPSDF5-mediated cell recruitment for IVD regeneration [19], we aim herein to explore the HAPSDF5 potential in vivo. First, we evaluated the IVD endogenous regenerative potential of HAPSDF5 intradiscal injection. Second, we explored IVD regeneration with HAPSDF5 injection combined with the intravenous injection of MSCs. Furthermore, we characterized the local and systemic inflammatory response associated with both strategies.

## 2. Results

**Figure 1:**
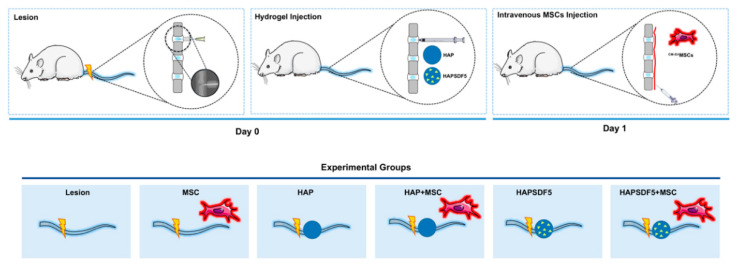
Experimental setup. An IVD needle injury was performed in rat caudal IVDs Co5/6, Co6/7, and Co7/8. Immediately after lesion induction, a hyaluronan-based hydrogel was administered in loco to the injured IVD with (n = 10) or without (n = 10) rat SDF-1. Lesioned only animals were kept as a control group (n = 10). For each of these groups, part of the animals (n = 5 each) had bone marrow MSC administered 24 h post-injury.

This study evaluated the therapeutic potential of a chemoattractant delivery system (HAPSDF5) that combines a hyaluronan-based thermoreversible hydrogel (HAP) and the chemokine stromal cell derived factor-1 (SDF-1) in IVD regeneration. HAPSDF5 was injected in the IVD and was combined with the intravenous injection of mesenchymal stem/stromal cells (MSCs) in a pre-clinical in vivo model of an IVD lesion. The local and systemic effects were evaluated two weeks after treatment. The experimental setup is detailed in Figure 1.

### 2.1. Potential of HAPSDF5 Intradiscal Injection to Recruit MSCs

**Figure 2:**
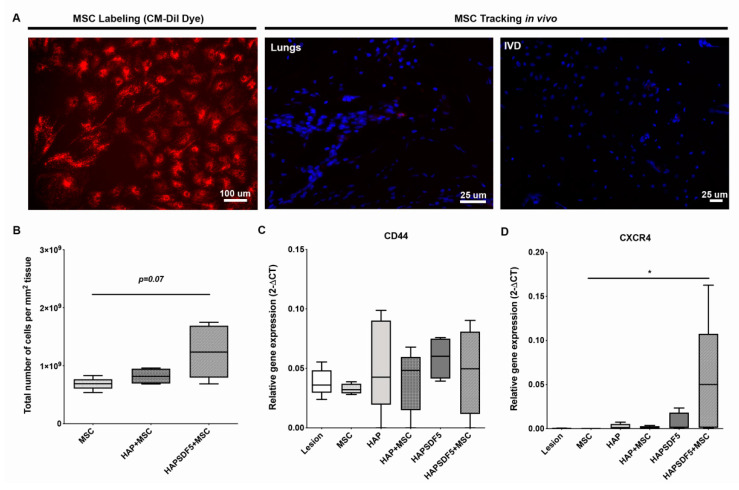
(**A**) rMSC labelling and in vivo tracking. On the left, rMSCs in culture 24 h after CM-DiI labelling (red); on the right, histological sections 2 weeks after administration showing presence of rMSCs in the lungs and the absence of rMSCs in IVD. (**B**) Total number of cells present in IVD identified by DAPI nuclear staining. (**C**,**D**) IVD gene expression analysis 2 weeks post-injury and intradiscal administrations for the hyaluronic acid receptor CD44 and the SDF-1 receptor CXCR4 normalized to GAPDH. Results are presented as box and whiskers plots (n = 4–5 animals). * *p* < 0.05.

MSC recruitment by SDF-1-containing HAP (HAPSDF5) was evaluated 2 weeks after IVD lesion induction by the presence of previously labelled CM-DiI-rMSCs (Figure 2A). No cells could be identified within or in the surroundings of the IVD. In contrast, few cells were identified in the lungs of the animals, suggesting that lung entrapment may have hampered the rMSC’s ability to reach the IVD tissue. As an indirect measurement of SDF-1 mediated cell recruitment to the IVD, the total number of cells present in the IVD was assessed by DAPI staining. There was an increase in the total number of cells in the HAPSDF5 group (HAPSDF5+MSC) compared to the control animals in which MSCs were injected but no HAP/HAPSDF5 was implanted in the injury site (*p* = 0.07) (Figure 2B). In order to access the IVD resident cell response to HAP and SDF-1 administration, the gene expression of CD44, the HA receptor, CXCR4, and the SDF-1 receptor were also addressed. An increase in CD44 was observed in the HAP administered groups, suggesting that HAP injection might impact its receptor expression by NP cells (Figure 2C). The same was observed for SDF-1 receptor CXCR4 gene expression, with a slight increase observed for the groups where SDF-1 was administered and with a significant increase for the HAPSDF5+MSC group in comparison to the MSC group (*, *p* < 0.05) (Figure 2D).

### 2.2. Potential of HAPSDF5 Intradiscal Injection for IVD Regeneration

**Figure 3:**
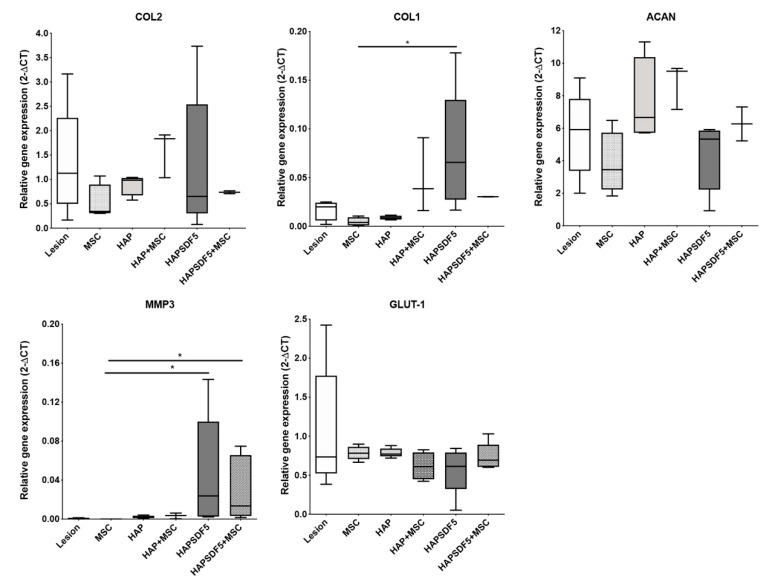
IVD gene expression analysis 2 weeks post-injury and intradiscal administrations. Relative gene expression of the main IVD ECM components: aggrecan (ACAN), collagen type I (COL1), collagen type II (COL2), matrix metalloproteinase MMP3, and oxidative stress marker GLUT-1, normalized to GAPDH. Results are presented as box and whiskers plots (n = 2–5 animals). * *p* < 0.05.

The effect of the intradiscal injection of HAPSDF5 was evaluated in the NP by the mRNA expression of selected genes, namely those related to the ECM and IVD degeneration (Figure 3), 2 weeks after treatment. The expression of collagen type I (COL1), collagen type II (COL2), and aggrecan (ACAN) revealed a great variation among groups, with a noticeable significant increase of COL1 expression in the group injected with the SDF-1 containing hydrogel (HAPSDF5) compared to the MSC injection group (*, *p* < 0.05). Matrix metalloproteinase (MMP) 3, a matrix metalloproteinase involved in ECM remodeling, and GLUT-1, a marker of oxidative stress, are also both increased in IVD degeneration. A significant increase in MMP3 expression (*, *p* < 0.05) was observed in the SDF-1-treated groups, suggesting a continuous and active ECM remodeling in the presence of this chemokine, while no alterations were observed for GLUT-1 (Figure 3).

**Figure 4:**
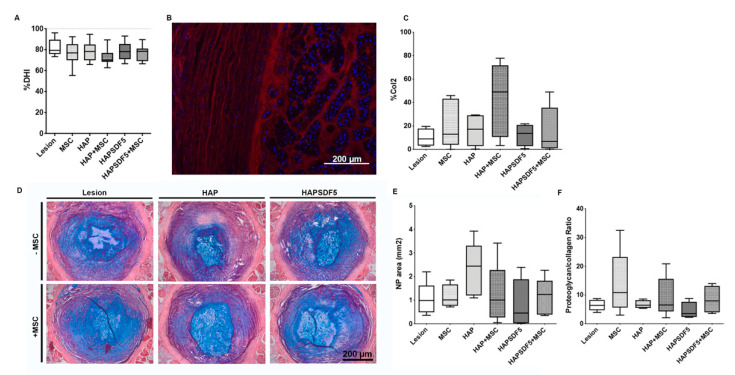
IVD radiological and histopathological analysis 2 weeks post-injury and intradiscal administrations. (**A**) Percentage of disc height index (%DHI) quantified from X-ray images. (**B**) Immunohistochemistry for collagen type II (Col II: red; DAPI for cell nuclei: blue, scale bar 200 µm) and (**C**) respective quantification. (**D**) Representative sections of alcian blue/picrosirius red staining (proteoglycans in blue, collagen in red) for groups lesion, lesion+MSC, HAP, HAP+MSC, HAPSDF5, and HAPSDF5+MSC (scale bar 200 µm); (**E**) quantification of the NP area (mm^2^); (**F**) quantification of the proteoglycans/collagen content ratio in the NP.

Moreover, the disc height index (%DHI) was calculated from an X-ray image of each animal before and after treatment. The results showed about a 20% decrease in the %DHI in all of the injured groups (with respect to the 100% expected in uninjured animals), but no significant difference was observed between treatments (Figure 4A).

The ECM content of the NP was also evaluated at the protein level by collagen type II (Col II) immunofluorescence (Figure 4B). The results showed increased Col II expression in the HAP+MSC group, which is indicative of a better outcome in terms of regenerative potential (Figure 4C). However, no differences were observed within the remaining treatments, which is in line with what was observed for the Col II gene expression levels. In addition, a detailed histological analysis was performed to evaluate the ECM content in the NP (Figure 4D). The NP area was first quantified based on alcian blue/picrosirius red proteoglycan staining. Although no statistically significant differences were observed between treatments, a slight increase was observed in the group containing the hydrogel alone (HAP) (Figure 4E). Moreover, no differences were observed in the proteoglycan/collagen ratio with any of the treatments. Still, it is clear from our results that there is an overall increase in the proteoglycan/collagen ratio in all of the groups that had been administered MSCs (Figure 4F).

### 2.3. Influence of Intradiscal HAPSDF5 Delivery in Hernia Formation and Local Immune Response

**Figure 5:**
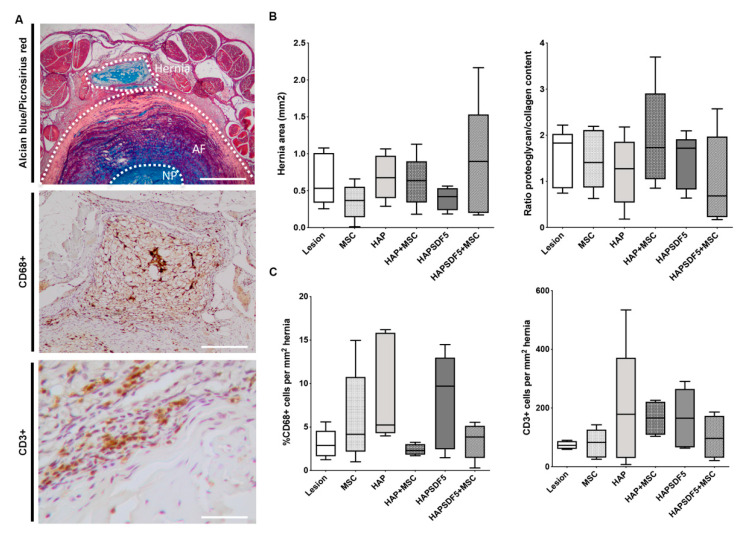
Hernia histopathological analysis 2 weeks post-injury and intradiscal administrations. (**A**) Representative images of the hernia with alcian blue/picrosirius red staining (hernia delimited by dashed line, scale bar 1 mm), macrophage identification within the hernia by CD68 IHC (positive cells are stained in brown, scale bar 200 µm), and T lymphocyte identification within the hernia by CD3 IHC (positive cells are stained in brown, scale bar 50 µm). (**B**) Quantification of the hernia area (mm^2^) across the depth of all of the sections of an IVD with visible herniation and proteoglycans/collagen content ratio in the hernia. (**C**) Percentage of CD68+ and CD3+cells within the hernia. Results are presented as box and whiskers plots (n = 5).

Following injury, beyond IVD degeneration, a pronounced hernia is formed, identified by the extrusion of proteoglycan-rich tissue, which in most cases, occurs at the region between the dorsal segmental muscles, as demonstrated in previous studies [34]. Hernia formation was assessed by alcian blue/picrosirius red staining and was quantified as previously described [34] by the delimitation of the hernia area in the histological sections (which comprises areas of proteoglycans-rich alcian blue staining). The results demonstrated similar hernia dimensions for all the groups, as well as a similar proteoglycan/collagen ratio in the hernia (Figure 5A,B); however, a slight decrease in hernia area was observed in the MSCs group, as previously reported [20]. Since hernia resorption has been linked to an inflammatory response and particularly to macrophage infiltration [35], the presence of macrophages and T-lymphocytes that had infiltrated the hernia was also analysed through the immunolocalization of CD68 and CD3, respectively. Generally, all of the groups presented infiltrates of both cell types, with a higher incidence in the group treated with the hydrogel alone (HAP) (Figure 5C). Interestingly, for the groups HAP+MSC and HAPSDF5+MSC, the systemic administration of MSCs seemed to reduce the number of macrophages and T-lymphocytes that were locally present at the hernia site.

### 2.4. Systemic Immune Response to Intradiscal HAPSDF5 Delivery

**Figure 6:**
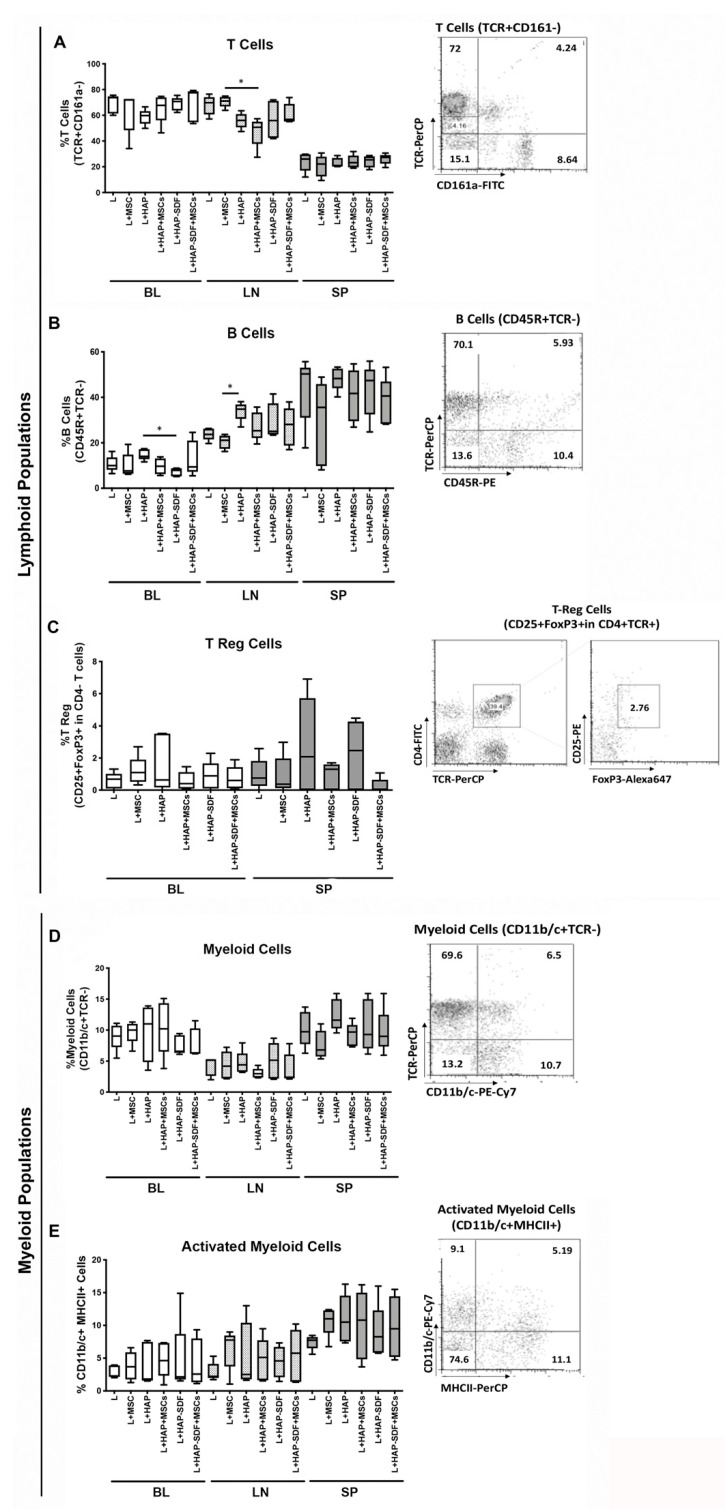
Systemic immune cell population profile. Phenotypic analysis of immune cell populations in peripheral organs: blood (BL), draining lymph nodes (LN), and spleen (SP), using flow cytometry. (**A**) T cells, (**B**) B cells, (**C**) Treg cells, (**D**) myeloid cells, (**E**) activated myeloid cells. Results are presented as box and whiskers plots. Representative plots illustrate the gating strategy and surface markers used to identify each cell population. * *p* < 0.05.

**Figure 7:**
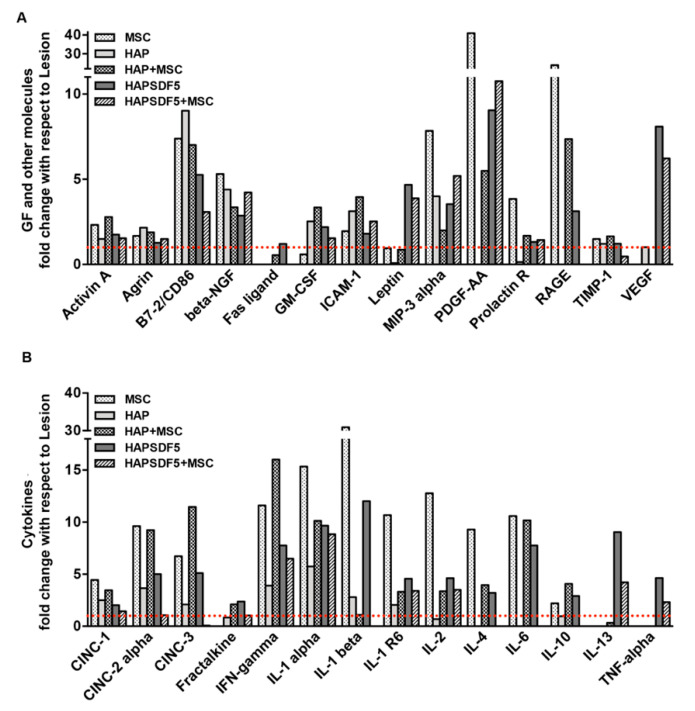
Plasma cytokines and growth factor profile. Semi-quantitative analysis of an array of inflammatory mediators in rat plasma expressed as fold-change with respect to the control lesion group (dashed line, y = 1). (**A**) Growth factors and other molecules; (**B**) cytokines. IFN-γ, MIP-3α, and IL-1β were not detected in the lesion group; for these, an arbitrary value of 1.5 was considered for the calculation of the fold-change for the remaining groups.

Along with the IVD local effect of HAPSDF5 administration, the systemic immune response was also investigated 2 weeks post-injury/treatment through the analysis of the immune cell populations in the blood (BL), draining lymph nodes (LN), and spleen (SP) (Figure 6) and through the assessment of cytokine/growth factor content in the plasma (Figure 7). Regarding immune cell populations and their activation status in the main systemic organs, we could observe a clear decrease in the proportion of T cells (TCR+CD161-) in the group with HAP+MSC in the LN but not in the BL and SP (Figure 6A). On the other hand, B cells (CD45R+TCR-) were increased in the LN for all of the groups containing the hydrogel with respect to the lesion and MSC groups, with differences reaching statistical significance for the HAP group when compared to the MSC group (Figure 6B). We went further in the analysis of the lymphoid cell population and evaluated the presence of Treg (CD25+ FoxP3+ in CD4+TCR+CD161a-) in the BL and SP of the animals. As expected, the results showed scarce Treg cell percentages that were slightly increased in the groups with HAP and HAPSDF5 in the SP compared to the lesion and MSC-related groups (Figure 6C). Regarding the analysis of the myeloid populations, a slight decrease in the myeloid cells (CD11b/c+TCR-) was observed in the HAP+MSC group in the LN and SP, compared to the HAP group (Figure 6D); no differences were found for any of the other groups. Moreover, we evaluated the frequency of the activated myeloid cells (CD11b/c+MHCII+ in Figure 6E), but no differences were observed between the groups.

Finally, the comparison of the plasma levels of more than 30 cytokines revealed that the groups containing HAPSDF5 (both with and without MSCs, had a high increase in the plasma levels of the growth factors leptin and the vascular endothelial growth factor (VEGF) (Figure 7A) and of the cytokines IL-13 and TNF-α (Figure 7B), compared to the other groups. No other obvious systemic effects were observed, associated with hydrogel administration.

## 3. Discussion

Cell homing to IVD has been explored as a promising regenerative medicine-based strategy. Although challenging due to its avascular nature, several studies have been able to demonstrate that during degeneration, the IVD can secrete chemokines that can efficiently attract progenitor cells, such as MSCs [8,36]. In vivo, we also successfully demonstrated the potential of systemically transplanted MSCs to prevent IVD herniation [20]. Despite the promising outcomes of MSCs, their harvesting from the bone marrow is an invasive procedure, and MSC expansion can be demanding and costly, other than the possible risks of MSC implantation in IVD, such as cell leakage and osteophyte formation [37]. We have shown that HAP hydrogels containing SDF-1 (HAPSDF5) significantly increased the recruitment of MSCs from the CEPs in an ex vivo IVD model [17], promoting faster and increased collagen type II expression [19]. Based on the idea of recruiting MSCs towards the degenerating IVD by locally increasing the concentration of a well-known chemoattractant, SDF-1, delivered by a HA-based thermoreversible hydrogel, this work aimed to demonstrate the in vivo potential of HAPSDF5 in (1) inducing endogenous cell recruitment; (2) recruiting intravenously transplanted MSCs; and (3) promoting IVD regeneration in a IVD lesion model [34].

Our results suggested SDF-1-induced cell migration, as noticed by an increased cell number in the group injected with HAPSDF5+MSC. We could not find any CM-DiI labeled rMSCs in the IVD or IVD surrounding tissues, suggesting that the increase in cell number may be due to endogenous cell recruitment from local niches. CM-DiI rMSCs were however found in the lungs after 2 weeks. Indeed, the migration of transplanted MSCs in several models is known to be critically impaired by the lung barrier, which hinders cell migration by entrapping cells in this organ [38]. Nonetheless, we found a significantly increased expression of CXCR4, the main SDF-1 receptor, in the HAPSDF5+MSC group only, suggesting that IVD cells only respond to SDF-1-hydrogel injection when MSCs are in circulation. Still, it must be disclaimed that the increased expression of SDF-1 and CXCR4 has previously been described in degenerated IVDs [25]. Others have proposed the increased expression of CD105 and CD90 in IVD as proof-of-concept for cell recruitment [29]. However, conclusions should be made carefully, as progenitor stem cells expressing these markers have also been previously described in the literature for the rat [39]. Finally, it must be noted that our study is centered at 2 weeks post-lesion, which may hinder the analysis of MSC recruitment towards the IVD and at the same time may be too early to observe clear IVD regeneration indicators, highlighting the constraints in developing combinatory therapies for the IVD.

The homing of MSC towards injury sites is known to be mediated by MMPs. In fact, we verified that MMP3 was significantly increased in the groups containing HAPSDF5, suggesting that ECM remodeling is happening in parallel with the SDF-1-induced migration of MSC [28]. Additionally, increased MMP3 expression at the gene and protein level has been suggested to facilitate endogenous stem cell homing through the remodeling of the cartilaginous endplate. Although positive, if linked to stem cell migration, the observed increase of MMP3 expression may also reflect an increased matrix breakdown as a consequence of a higher degree of degeneration [40], which was also suggested by the increased expression of COL1 in the HAPSDF5 group. Contrary to previous data [20], neither the MSC transplantation, nor the treatment with HAPSDF5 were able to increase disc height, showing values very similar between different groups. The most relevant changes observed in the ECM consist of a slight increase of the proteoglycan/collagen ratio and of Col II expression at the gene and tissue level in all of the groups treated with MSC transplantation. This emphasizes the regenerative potential of MSCs through their paracrine effect, regardless of MSC recruitment to the site of the lesion. On the other hand, both groups injected with HAPSDF5 (with and without MSC) had the lowest proteoglycan content, indicating a poor regenerative prognosis. By observing the local response, we noticed that hernia areas did not show differences within the different groups but also that a higher infiltration of immune cells (macrophages and T-lymphocytes) was observed in the groups without MSC, suggesting that systemic injection of MSCs might decrease the local immune response. This is particularly interesting with the knowledge that SDF-1 is also chemotactic for lymphocytes and macrophages [41].

Regarding the systemic immune response, namely the characterization of immune cell populations and their activation status in the main systemic organs, we could observe that HAP administration in the IVD resulted in a clear decrease in T cells and an increase in B cells in the LN as well as in an increase in the Tregs in the SP. Interestingly, but not surprisingly, the groups in which the MSCs were administered (HAP+MSC and HAPSDF5+MSC) reduced the Tregs to lesion levels, demonstrating once again the immunomodulatory potential of MSCs [20]. Additionally, it is interesting to notice that the local administration of HAP in IVD resulted in increased T cells in the hernia site and decreased T cells at the systemic level in the draining LN. Systemic inflammatory response seems to be in contrast with the local inflammatory response. This dynamic nature of the immune response to the IVD lesion at the organism level has been previously reported upon in the same model at the same time point post-injury [42]. In addition, higher systemic levels of VEGF were found in the HAPSDF5 and HAPSDF5+MSC groups. In fact, SDF-1 has been described as regulating a distinct macrophage differentiation program, characterized by the expression of angiogenic factors such as VEGF and CCL1 [41]. Systemic levels of leptin were also increased in the HAPSDF5 groups (with and without MSC). Although unexpected, the presence of higher levels of this hormone might be related to its involvement in the signalling of MSC survival and chemotaxis under low oxygen levels [43].

One of the main limitations of this study was the time selected for both MSC recruitment and IVD analysis since two weeks is probably a very long time to evaluate cell recruitment upon intravenous injection, but it is an appropriate time to evaluate IVD herniation in this model [34]. Additionally, another limitation is the lack of knowledge on the different cell types recruited by the HAPSDF5 hydrogel, which could be further explored in detail in the future by immunofluorescence analysis using different antibodies.

Overall, HAPSDF5 was ineffective in thwarting the induced disc degeneration, while the MSCs seem to exert a role in IVD regeneration at a distance through a paracrine effect. Further studies with longer time points or in vivo models with less invasive IVD lesions might be required to address the full potential of this chemoattractant delivery system.

## 4. Materials and Methods

### 4.1. IVD Lesion Model

Male Wistar Han (Crl:WI/Han) rats (30 rats, n = 5/group) at 2 months of age were used for the IVD caudal injury model, as previously described [34] (Figure 1). Briefly, the animals were anaesthetized by means of isoflurane inhalation, placed in prone position, and the tail skin was disinfected with ethanol. A 21-G percutaneous puncture was performed in the coccygeal IVDs Co5/6, Co6/7, and Co7/8 using radiography for IVD identification. Experiments were conducted at the i3S-Instituto de Investigação e Inovação em Saúde animal facility, and approved by the i3S Animal Welfare and Ethics Review Body and the Portuguese Competent Authority (DGAV) through license n° 3773/2015-02-09 and were conducted in accordance with the European Legislation on Animal Experimentation through Directive 2010/63/UE.

### 4.2. Hydrogel Preparation and SDF-1 Incorporation

HA sodium salt from streptococcus equi was purchased from Contipro Biotech s.r.o. (Dolní Dobrouč, Czech Republic) with an average molecular weight of Mw = 1.5 MDa and a polydispersion index = 1.53. Amino-terminated poly(*N*-isopropylacrylamide) (pNIPAM-NH2) of 44 ± 2.7 kDa was purchased from Polymer Source, Inc. (Dorval, QC, Canada). HAP was prepared by a direct amidation reaction of the thermoreversible segments of pNIPAM-NH2 on the HA backbone, as previously described [33]. The polymer solution was filter-sterilized, lyophilized, and then reconstituted in sterile phosphate-buffered saline (PBS) (pH 7.4) at a concentration of 10% wt/vol (HAP) or additionally with the incorporation of rat chemokine stromal cell-derived factor 1 (SDF-1) (Peprotech, London, UK) at a concentration of 5 ng/µL (HAPSDF5).

### 4.3. Hydrogel Intradiscal Administration

Immediately after generating the lesion, the Co5/6, Co6/7, and Co7/8 discs were submitted to the intradiscal administration of 10µl of the hydrogel with (n = 10) or without (n = 10) rat SDF-1 at 5 ng/µL using a 29-G insulin needle attached to a handmade adaptor to assure a 5 mm depth administration to the center of the IVD (Figure 1). All material was kept at 4 °C to avoid hydrogel jellification prior to administration. Lesion-only animals were kept as controls (n = 10).

### 4.4. rMSC Isolation, Labelling and Administration

Rat bone marrow MSCs (rMSC) were isolated from the femoral and tibial bone marrow. rMSCs were cultured in α-MEM medium supplemented with 10% FBS (Thermo Fisher Scientific, Waltham, MA, USA) and were expanded up to P3. rMSCs phenotype was characterized using flow cytometry as CD29+, CD90+, and CD45-, as previously performed [20]. A total of 24 h prior to in vivo administration, cells were incubated with 10 µM CellTracker CM-DiI (Invitrogen, Carlsbad, CA, USA) for 5 min at 37 °C followed by 15 min at 4 °C. Cells were then washed in 1x PBS and were incubated in fresh medium. All cell cultures were prepared at 37 °C in an atmosphere of 5% CO_2_, and cell handling procedures were performed in a sterile laminar flow hood. For systemic transplantation, 1 × 10^6^ cells at P3 were resuspended in 400 µL of sterile saline solution and administered by intravenous injection into the lateral tail vein using a 24-G catheter (Braun, Frankfurt, Germany) under anaesthesia, as previously performed [2,20] (Figure 1). For each of these groups (lesion and groups with HAP and HAPSDF5), n = 5 animals per group had bone marrow MSCs administered 24 h post-injury.

### 4.5. Determination of the Disc Height Index

Digital radiographs were acquired by the Owandy-RX radiology system equipped with an Opteo digital sensor (Owandy Radiology, Croissy-Beaubourg, France) and were processed with QuickVision software (Owandy Radiology, Croissy-Beaubourg, France). The percentage of the disc height index (% DHI) was calculated using ImageJ 1.47i software (Bethesda, MD, USA) for radiograph measurements, as previously described [34].

### 4.6. IVD Collection and Histological Analysis

Target IVDs with adjacent vertebrae were collected 2-weeks’ post-injury and were fixed in 10% neutral buffered formalin (Bio-Optica, Milan, Italy) for 1 week at room temperature. Tissue was decalcified in an EDTA-glycerol solution and was processed for paraffin embedding. Sequential transversal 5-µm sections of the IVD were collected. Sections were deparaffinized in xylene, rehydrated through a graded series of ethanol, and processed for alcian blue/picrosirius red staining, immunofluorescence for collagen type II, and immunohistochemistry for CD68 and CD3.

### 4.7. Alcian Blue/Picrosirius Red Staining

Rehydrated sections were incubated in Weigert’s Iron Hematoxylin for 10 min, washed in tap water, and stained in alcian blue solution pH 2.5 for 30 min. After rinsing in tap water, sections were incubated in picrosirius red solution (0.1 g sirius red in 100 mL of saturated aqueous picric acid solution) for 1 h, followed by being washed in 0.01 N HCl for 2 min. Sections were dehydrated, mounted with Entellan (Merck Millipore, Burlington, MA, USA), and analysed in a CX31 optical microscope equipped with a DP25 digital colour camera (Olympus, Hamburg, Germany). The hernia and NP area was determined by delimitating regions of interest (ROI) in each optical section, considering blue staining for proteoglycans and red staining for collagen. The hernia area was calculated as the mean of the areas of each individual section throughout the IVD as previously described [34]. Within the hernia and NP ROIs, the % area of the proteoglycans and collagen was determined by a custom ImageJ macro based on a colour deconvolution technique used to separate the different colour channels [44].

### 4.8. Immunofluorescence for Collagen Type II

Collagen type II (Col II) distribution in the IVD was analysed by immunofluorescence staining. Antigen retrieval was performed in paraffin sections through incubation with 20 μg/mL proteinase K (Sigma-Aldrich Inc., St Louis, MO, USA) solution for 15 min at 37 °C. Sections were incubated overnight at 4 °C with the anti-collagen II-II6B3 (Developmental Studies Hybridoma Bank, 1:20 dilution) antibody followed by incubation with Alexa Fluor 594-labeled goat anti-mouse antibody (1:1000, Molecular Probes, Invitrogen, Carlsbad, CA, USA). Sections were mounted in Fluorshield with DAPI (Sigma-Aldrich Inc., St Louis, MO, USA). Representative images of the slides were collected using an inverted fluorescence microscope (Axiovert 200 M, Zeiss, Jena, Germany). The %COL2 in the NP was determined as previously described [20].

### 4.9. Immunohistochemistry for CD68+ and CD3+ Cells

Immunohistochemistry (IHC) for the detection of CD68+ and CD3+ cells in the hernia was performed by the Novolink^TM^ Polymer Detection Kit (Leica Biosystems, Newcastle, UK) following the manufacturer’s instructions. Antigen retrieval was performed through incubation in near-boiling point 10 mmol/L sodium citrate buffer with a pH of 6.0 for 1 min, followed by incubation with 20 μg/mL proteinase K (Sigma-Aldrich Inc., St Louis, MO, USA) solution for 15 min at 37 °C. Sections were incubated with anti-CD68 (clone ED1, 1:100 dilution, Bio-Rad Laboratories, Irvine, CA, USA) or anti-CD3 (undiluted, Leica Biosystems, Newcastle, UK) antibodies overnight at 4 °C. The stained sections were imaged with light microscopy. CD3+ and CD68+ cells were quantified using ImageJ tools directly on the acquired images. From these, the %CD3 positivity was normalized to the total number of cells in the hernia area, and the %CD68 positivity was normalized to the area of the hernia.

### 4.10. RNA Isolation and Quantitative Real-Time Reverse Transcription Polymerase Chain Reaction

Total RNA was isolated from the NP using TRIzol reagent (Ambion Inc, Waltham, MA, USA) and was quantified by Nanodrop spectrophotometry (Thermo Fisher Scientific, Waltham, MA, USA). The high-capacity cDNA reverse transcription kit was used per the manufacturer’s instructions (Applied Biosystems, Waltham, MA, USA). Gene expression levels were determined by qRT-PCR conducted on an iQ5 Real-Time PCR Detection System (Bio-Rad Laboratories, Irvine, CA, USA) using the TaqMan Gene Expression Master Mix and TaqMan Gene Expression Assays (Applied Biosystems, Waltham, MA, USA) for aggrecan (Rn00573424_m1), collagen type I (Rn01463848_m1), collagen type II (Rn01637087_m1), MMP3 (Rn00591740_m1), GLUT-1 (Rn01417099_m1), CXCR4 (Rn00573522_s1), CD44 (Rn00681157_m1), and glyceraldehyde 3-phosphate dehydrogenase (GAPDH, Rn99999916_s1) as reference genes. The quantification cycle (Cq) 35 cutoff was used. Relative expression levels were calculated using the Cq method (∆Ct = Ct(_gene of interest_ − Ct(_GAPDH_)) according to published guidelines.

### 4.11. Plasma Cytokine Quantification

The membrane-based Rat Cytokine Antibody Array C2 (RayBiotech, Calgary, AB, Canada) was used for the semi-quantitative detection of cytokines in the plasma, according to the manufacturer’s instructions. The plasma pool (n = 5) for each group was analysed. The signal density for each sample spot was determined using Chemidoc XRS+ (BioRad Laboratories, Irvine, CA, USA) and ImageJ software. Relative cytokine levels were normalized to the positive internal control group and to the lesion group. IFN-γ, MIP-3α, and IL-1β were not detected in the lesion group, so for these, an arbitrary value of 1.5 instead of 0 was considered for the calculation of the fold-change.

### 4.12. Flow Cytometry Analysis of Systemic Immune Cell Populations

Animals were maintained under general anesthesia with volatile isoflurane, and whole blood (BL) was collected by means of a cardiac puncture into a 1:10 anticoagulant citrate–phosphate–dextrose solution with adenine (Sigma-Aldrich Inc., St Louis, MO, USA). The animals were then dissected for collection of draining iliac and inguinal lymph nodes (LN) and spleen (SP). BL, LN, and SP were immediately processed for flow cytometry analysis.

After blood centrifugation at 800× *g* for 20 min at room temperature, the plasma and buffy coats were separately collected. The plasma was further centrifuged twice at 2500× *g* for 15 min at 4 °C to remove cell debris and was kept at −80 °C for cytokine analysis. The collected buffy coats were diluted with PBS, overlaid on Lymphoprep (Axis-Shield Diagnostics, Dundee, Scotland) in a 1:1 ratio, and centrifuged at 800× *g* for 30 min at room temperature, without a break, in order to isolate peripheral blood mononuclear cells (PBMC). Lymph node cells were isolated through the mechanical dissociation of the lymph nodes on a 100-μm pore cell strainer. Spleen cells were collected by a similar process, injecting 100 U/mL Collagenase type I (Sigma-Aldrich Inc., St Louis, MO, USA) before mechanical dissociation.

The red blood cells in the spleen cell suspension were then lysed by incubation with NH_4_Cl 150 mM in Tris 10 mM solution for 8 min at 37 °C. Cell surface staining for flow cytometry was performed in 96-well plates in FACS buffer (0.5% BSA, 0.01% sodium azide, PBS) for 30 min on ice after Fc receptor blocking. The following anti-rat antibodies were used: CD45R-PE (clone HIS24), TCR-PerCP (clone R73), major histocompatibility complex class II (MHCII)-PerCP (clone OX-6), CD4-APC (clone OX35), and CD161a-FITC (clone 10/78), CD11b/c-PE-Cy7 (clone OX-42), and CD25-PE (Clone OX-39) (BD Biosciences, San Jose, CA, USA) and FoxP3-Alexa647 (Clone 150D) (BioLegend, San Diego, CA, USA). Samples were acquired on a flow cytometer (FACSCanto II; BD Biosciences, San Jose, CA, USA), and data were analyzed with FlowJo software version 8.7 (FlowJo, Ashland, OR, USA).

### 4.13. Statistical Analysis

The results are presented as median ± interquartile range (IQR) in box and whiskers plots. Normality was assessed with the D’Agostino–Pearson omnibus normality test. Statistical analysis was performed with the non-parametric Kruskal–Wallis test followed by Dunn’s multiple comparison test using GraphPad Prism 7. Statistical significance was set at * *p* < 0.05.

## 5. Conclusions

This work provides a comprehensive in vivo analysis of the HAPSDF5 chemoattractant delivery system for IVD tissue engineering in a small animal model of an IVD lesion. We demonstrated that a hyaluronan-based thermoreversible hydrogel (HAP) has the potential to be used for the intradiscal administration of biological therapeutics and that SDF-1 induces some degree of cell migration towards the IVD lesion. However, no evidence of IVD regeneration was found in response of HAPSDF5 despite its promising results ex vivo. This study highlights the importance of performing pre-clinical in vivo studies for IVD regeneration as well as of understanding the limitations and clinical scenarios for cell-recruitment strategies for IVD.
